# Factor Structure, Construct Validity, and Measurement Invariance of the Neuroception of Psychological Safety Scale (NPSS)

**DOI:** 10.3390/ejihpe14100178

**Published:** 2024-10-04

**Authors:** Marta Spinoni, Andrea Zagaria, Anna Pecchinenda, Caterina Grano

**Affiliations:** Department of Psychology, Sapienza University of Rome, 00185 Rome, Italy; marta.spinoni@uniroma1.it (M.S.); anna.pecchinenda@uniroma1.it (A.P.); caterina.grano@uniroma1.it (C.G.)

**Keywords:** psychological safety, polyvagal theory, neuroception, measurement invariance, psychometric properties, cross-cultural adaptation

## Abstract

Psychological safety has recently emerged as a central construct, strictly implicated in mental health and emotional well-being. The Neuroception of Psychological Safety Scale (NPSS) is the first scale designed to assess feelings of psychological safety from a multidimensional perspective. However, the robustness of its factorial structure requires further examination in large community samples, and evidence of construct validity along with measurement invariance across genders is scarce. The present study aimed to address these gaps through a comprehensive cross-validation approach. A community sample of 660 Italian adults, aged 18–65, completed self-report questionnaires including the NPSS, the Compassionate Engagement and Action Scale (CEAS), the Sussex-Oxford Compassion for Others (SOCS), and the Body Perception Questionnaire (BPQ). A three-factor model, i.e., *Social Engagement*, *Compassion*, and *Bodily Sensations*, demonstrated a good fit to the data in two random subsamples. Moreover, the measurement model was factorially invariant across genders. Model-based omega coefficients supported the internal consistency of the NPSS scores (ω ranged between 0.879 and 0.918). Zero-order correlations between NPSS subscales and CEAS, SOCS, and BPQ provided construct validity evidence. Additionally, inter-correlations between latent factors and Harman’s single-factor test supported the discriminant validity of the NPSS dimensions. Overall, this study provided compelling evidence regarding the psychometric properties of the NPSS, demonstrating for the first time the invariance of its factorial structure across gender.

## 1. Introduction

Psychological safety has recently emerged as a central construct, strictly implicated in mental health and emotional well-being [1,2,3]. It refers to individual perceptions of safety within interpersonal relationships and environmental contexts [4] and, by promoting physiological states of rest, is pivotal for social connection and behaviors [3]. In psychologically safe environments, individuals feel secure enough to express their thoughts, ideas, and concerns without fearing negative consequences, such as humiliation or shame [5]. Moreover, early secure and caring experiences may shape the nervous system and foster the development of adaptive regulation strategies that individuals can rely on later in life [6,7]. Conversely, an unregulated threat environment can affect biological processes, development, and social interactions, leading to trauma symptoms [8,9]. Despite the growing recognition of psychological safety as a crucial factor in individual well-being, the availability of psychometrically sound measures is limited. Existing research largely focuses on related constructs such as emotional safety or trauma, but few studies have operationalized psychological safety as a distinct concept or developed robust tools for its measurement [10,11].

The polyvagal theory (PVT), rooted in neurophysiology, psychology, and evolutionary theory, provides a comprehensive explanation of psychological safety by describing how situations are subconsciously assessed for safety or threat through “neuroception” [10,11], which refers to the neural processes responsible for detecting cues in the environment influencing individuals’ psychological, physiological, and emotional response [3,11]. According to this theory, situations and environments perceived as unsafe trigger defensive survival strategies, leading to immobilization, fight-or-flight response, or dissociation. In contrast, situations perceived as safe activate processes that promote social engagement, enhancing compassion and interpersonal connection [12]. In particular, previous research has underscored the significance of feeling safe within mental health contexts, revealing that perceived safety is negatively correlated with levels of depression, anxiety, stress, and self-criticism [13]. Furthermore, a sense of safety during hospitalization has been linked to increased feelings of control, calm, and hope [14] and has been shown to facilitate better recovery and healing [15].

Given its recent emergence as an innovative construct with substantial implications for mental health, psychological safety has garnered increasing interest in scientific literature. Importantly, since interpersonal safety is often communicated through compassion, employing interventions such as soothing voice tones and controlled breathing can reduce the fight-or-flight response (e.g., reduce the heartbeat), facilitate parasympathetic rest and repair, and subsequently promote psychophysiological well-being [16,17]. Consistently, interventions prioritizing the feeling of safety, such as the ‘Seeking Safety’ treatment for PTSD, have demonstrated efficacy in symptom reduction and psychiatric distress [18,19].

Various psychological scales have been developed to evaluate feelings of psychological safety, emphasizing its importance in diverse contexts, including organizational contexts and teamwork [20], medical settings, and trauma recovery [21,22] and in challenging situations [23]. Among these, drawing on the foundations of PVT, the Neuroception of Psychological Safety Scale (NPSS) has been recently developed [11,24]. The NPSS is a 29-item self-report measure specifically designed to assess individual neuroceptive processes underlying psychological safety in the context of individual experiences and interpersonal relationships [24]. The scale was developed by experts in polyvagal theory and therapists working with trauma after asking them what it meant to ‘*feel safe*’. The authors initially conducted an Exploratory Factor Analysis (EFA) considering 74 items and a polychoric parallel analysis to explore the factor structure, revealing a 3-factor model including the dimensions of Compassion, Social Engagement, and Bodily Sensations [24]. The Cronbach’s alpha coefficients of the subscales demonstrated high internal consistency [24]. However, some limitations in the original validation study need to be further addressed. Specifically, the Confirmatory Factor Analysis (CFA) showed that the proposed 3-factor model did not approach the recommended cutoff levels for satisfactory fit [25,26]. For instance, the Comparative Fit Index (CFI) and the Tucker–Lewis Index (TLI) were 0.86 and 0.84, respectively, both below the recommended thresholds for acceptable (0.90) or good (0.95) relative fit [25,26]. Moreover, no research has yet explored the gender measurement invariance of the NPSS within any cultural context. We deem that analyzing factorial invariance is essential since previous studies have identified significant gender differences in constructs closely related to psychological safety, such as compassion [27] and interoception [28], and given the well-established gender differences in mental health [29,30]. Ensuring the absence of differential item functioning between males and females is therefore crucial for facilitating an unbiased comparison of factor means in the assessment of psychological safety. Recently, Poli and Miccoli [31] further investigated the factorial structure of the instrument in a sample of 330 Italians from the general population, confirming the three dimensions identified in the original validation study [24]; however, factorial invariance across gender was not tested.

With this goal in mind, the present study aims to address the aforementioned gaps, providing further evidence regarding the dimensionality, reliability, construct validity, and gender invariance of the NPSS. More specifically, we first aimed to examine the robustness and replicability of the original factorial structure of the scale proposed by Morton et al., using a cross-validation approach [32]. That is, we expected that the three dimensions of *Social Engagement*, *Compassion and Bodily Sensations* adequately reproduced the inter-item correlations. Secondly, the study aims to provide additional evidence concerning the construct validity of the scores. Specifically, the following hypotheses were examined through correlation analyses: (a) The Social Engagement factor of the NPSS will exhibit a positive correlation with a convergent measure of compassion from others. Indeed, given that Social Engagement refers to the prosocial aspects of interacting with others, including behaviors such as connecting, communicating, and showing curiosity, and is characterized by feelings of being accepted, understood, cared for, and the freedom to express oneself without fear of judgment, it is expected to align strongly with scales assessing the perception of compassion received from others. (b) The Compassion factor of the NPSS will be positively correlated with a convergent scale that measures compassion for others. The Compassion factor captures the ability to feel and express compassion, including aspects of empathy, care, and the desire to help others, and reflects an individual’s capacity for emotional connection and supportive responses to others’ distress, making it conceptually similar to measures of compassion towards others. (c) The Bodily Sensations factor will be correlated with a measure of interoceptive sensibility (i.e., awareness of one’s body perceptions). The Bodily Sensations factor encompasses internal physiological states associated with calm and relaxation, such as a steady heartbeat, regular breathing, and a settled stomach. This factor reflects the individual’s ability to perceive and interpret internal bodily signals, aligning with constructs of interoceptive awareness, which is crucial for understanding physiological responses to stress and maintaining homeostasis. By examining these correlations, the study aims to provide additional evidence of the NPSS construct validity, demonstrating how its factors relate to established measures of compassion and interoceptive sensibility.

Thirdly, we aimed to investigate the measurement invariance of the NPSS across gender. Ensuring factorial invariance is pivotal to ascertaining that the scale’s latent dimensions maintain consistent meaning for both males and females, thus ensuring that any score difference can be accurately interpreted as true gender differences. Hence, it is crucial to ensure the absence of differential item functioning across males and females to guarantee an unbiased comparison of (latent) factor means [33].

## 2. Material and Methods

### 2.1. Participants and Procedures

The sample size was determined using an RMSEA-based power calculation for the test of reasonable fit on the three-factor structure found by Morton and colleagues [24]. For a power of 0.80, fixing the critical alpha to 0.05, the minimum sample size needed was 77 (df = 374). Moreover, at least 10 subjects per indicator were guaranteed according to common rules of thumb [34]. In view of the above, we recruited a sample of 660 Italian adults, aged 18–65 (M = 35.54, SD = 14.63; 58.3% females).

Participants in the present study were collected through social media platforms and were asked to answer an online anonymous questionnaire lasting around 15 min. Participants need to reside in Italy and be Italian citizens above 18 of age. They took part voluntarily and were not remunerated. After explaining the procedure of the study and signing the informed consent form, respondents were asked to complete a group of self-report questionnaires through an online survey hosted by the Qualtrics platform (https://www.qualtrics.com/; accessed on 01 August 2024). Anonymity was guaranteed by using alphanumeric codes. The socio-demographic characteristics of the sample are summarized in Table 1.

### 2.2. Measures

#### 2.2.1. Neuroception of Psychological Safety Scale (NPSS)

The Neuroception of Psychological Safety Scale (NPSS) was used to assess multiple dimensions of psychological safety [24]. The items are rated on a 5-point Likert scale from “1” (*strongly disagree*) to “5” (*strongly agree*), resulting in three composite scores, each one for the three subscales. In particular, a higher score in the Social Engagement factor indicates an evaluation of the social environment as non-threatening and safe to engage socially. The second factor captures the ability to be compassionate and feel connected, caring, and empathetic. The third factor is related to the internal sensations of the body in a state of calm. Examples of items from each subscale are, respectively: “*I felt accepted by others*”, “*I felt like* “*I could comfort a loved one*” and “*Breathing felt effortless*”.

For the purpose of this study, following international guidelines for psychological test adaptation [35,36], the NPSS was adapted into Italian following a five-stage procedure. Firstly, the NPSS was independently translated from English into Italian by two experts fluent in both languages and with expertise in the field of psychological safety. The two versions were synthesized into one common Italian version, and any discrepancies were resolved through consensus. The retained Italian version was backtranslated into English by an independent bilingual researcher who ignored the original scale composition. To verify the equivalence of the original scale and the back-translated English version, an independent researcher expert in the field compared the two scales. Eventually, the Italian version of the NPSS was administered in a pilot study of 20 participants to verify the comprehensibility of the contents. The participants rated the comprehensibility of each item on a 5-point scale (1 = do not understand at all; 5 = understand completely). The average ratings per item were examined, and since all items received a satisfactory rating (>4), no further revisions were implemented.

#### 2.2.2. Compassionate Engagement and Action Scale (CEAS)

To assess the construct validity of the Social Engagement subscale of the NPSS, the Compassion from Others—part A (Engagement) subscale of the Compassionate Engagement and Action Scale [16] was administered. This subscale of the CEAS consists of 8 items assessing the compassion people experience from others, with a high score reflecting high perceived compassion. Examples of items are “*Others are accepting, non-critical, and non-judgmental of my feelings of distress*” and “*Others tolerate my various feelings that are part of my distress*”. Cronbach’s alpha in the present investigation was 0.85.

#### 2.2.3. Sussex-Oxford Compassion for Others (SOCS-O)

To assess the construct validity of the Compassion subscale of the NPSS, the Sussex-Oxford Compassion for Others (SOCS-O) [37] was employed. The SOCS-O is a self-report measure comprising 20 items assessing compassionate behavior and feelings toward others. Examples of items are “*I recognize when other people are feeling distressed without them having to tell me*” and “*I’m quick to notice early signs of distress in others*”. The Italian adaptation of the SOCS-O was administered [38], and Cronbach’s alpha in the present sample was 0.91.

#### 2.2.4. Body Perception Questionnaire (BPQ)

To assess the construct validity of the Body Sensation subscale of the NPSS, the Body Awareness subscale of the Body Perception Questionnaire (BPQ) [39] was administered. This subscale is a 26-item self-report instrument evaluating individuals’ subjective experience of information arising from within the body. An example of an item is “*I feel my mouth is dry*”, evaluated on a 5-point Likert scale from 1 (never) to 5 (always). Higher scores correspond to higher levels of negative body-related sensation awareness. The Italian adaptation of the BPQ showed satisfactory psychometric properties [40], and Cronbach’s alpha in the present investigation was 0.93.

### 2.3. Data Analysis

Data were analyzed using IBM SPSS v. 25 [41] and Mplus v. 8.6 [42].

First, item-level descriptive statistics and Mardia’s [43] multivariate normality tests were calculated. In order to reduce the probability of capitalizing on chance solutions stemming from sampling error [32,44], a cross-validation approach was employed to analyze the factorial structure of the NPSS by randomly splitting the total sample into two halves (i.e., calibration and validation samples). The random split was performed using IBM-SPSS facilities. In the first subsample (i.e., calibration sample), the three-factor CFA solution originally proposed by Morton and colleagues [24] was estimated, and diagnostic information was analyzed to uncover potential sources of model misspecification [44]. Thereafter, to prevent the possibility that model modifications were due to chance variations [45], the resulting factor solution was cross-validated on the second subsample (i.e., validation sample). To establish the discriminant validity of the emerging dimensions, inter-factor correlations were analyzed [44] and a competitive one-factor model was estimated (i.e., Harman’s single-factor test) [46]. Inter-factor correlations lower than 0.80 and a poor fit to the data of the one-factor model provide support for the distinctiveness of the latent dimensions [44,47]. Due to significant departures from multivariate normality and the ordered categorical nature of the items [48], CFAs were fitted to the inter-item polychoric correlation matrix using the weighted least squares mean and variance-adjusted estimator (WLSMV) [42]. The goodness of fit of the models was assessed through multiple indices [26,49], including the root mean square error of approximation (RMSEA; ≤0.08 indicates a reasonable fit), the comparative fit index and Tucker–Lewis index (CFI and TLI, respectively; ≥0.90 indicates acceptable fit), and the standardized root mean squared residual (SRMR; ≤0.08 indicates acceptable fit). The χ^2^ test statistic was also reported, acknowledging its well-known dependence on sample size (e.g., [50]).

Model-based internal consistency of the NPSS was estimated via omega coefficients using Green and Yang’s [51] formula for ordinal items, with the model-implied variance of the score in the denominator to account for potential error covariances [52]. Proofs of construct validity were gathered through zero-order correlations between the NPSS, CEAS, SOCS-O, and BPQ. Specifically, the following hypotheses were examined through correlation analyses: (a) the Social Engagement factor of the NPSS will be positively correlated with the Engagement subscale of the CEAS; (b) the Compassion factor of the NPSS will be positively correlated with SOCS-O scores; and (c) the Bodily Sensations factor will be negatively correlated with the Awareness subscale of the BPQ. According to Cohen’s conventions [53], correlation coefficients higher than 0.10 are interpreted as small, higher than 0.30 as moderate, and higher than 0.50 as large.

Finally, factorial invariance tests across gender were conducted through multi-group CFA and following the framework proposed by Meredith [54]. We started with a configural invariance model by examining the same number of factors and the same pattern of free and fixed loadings across groups. Thereafter, we introduced equality constraints on factor loadings and intercepts, testing for metric and scalar invariance, respectively. To assess whether the hypothesis of parameter invariance can be retained, differences in goodness of fit indices were calculated, where ΔCFI ≥ 0.010 accompanied by ΔRMSEA ≥ 0.015 suggests a significant decrease in model fit [55,56].

## 3. Results

### 3.1. Preliminary Analyses

Item-level descriptive statistics are reported in Supplementary Table S1. Mardia’s [43] multivariate skewness and kurtosis coefficients indicated significant deviations from a multivariate normal distribution (*p* < 0.001), precluding the use of a standard maximum likelihood estimator, as it assumes that the sample covariance matrix is obtained from continuous normally distributed variables [48]. Therefore, and considering that NPSS items are observed on an ordinal scale of measurement (e.g., [48,57]), the WLSMV estimator was employed for further CFAs [42], fitting the model to the polychoric inter-item correlations.

### 3.2. Factor Structure

Following Morton and colleagues [24], a three-factor CFA was specified on the first subsample showing an unsatisfactory fit to the data: χ^2^ (374) = 1306.920, *p* < 0.001; CFI = 0.916; TLI = 0.909; RMSEA = 0.087 (90% CI 0.082–0.092); and SRMR = 0.060. An examination of the modification indices provided evidence for correlated residuals between *Item 4* (“*I felt understood*”) and *Item 5* (“*I felt like others got me*”), and between *Item 7* (“*There was someone who made me feel safe*”) and *Item 8* (“*There was someone that I could trust*”). The inspection of the wording and content of the Italian translation of the NPSS suggested that there were substantial similarities among the items. Differently from the other set of indicators, *Items 7* and *8* are very similarly worded [44], which, along with the proximity, may increase their shared variance above and beyond the latent factor [58]. Moreover, *Items 7* and *8* suffer from content overlap [59] and are positioned adjacent in the scale (i.e., proximity effect; [58]); thus, it was reasonable to hypothesize that some of their covariation was due to sources other than the common factor. In order to preserve the content validity of the scale and cover all relevant elements of the construct it aims to represent, items were retained, and the CFA was re-specified freely estimating the error covariances between these pairs of indicators (i.e., Item 4 with Item 5, and Item 7 with Item 8). The revised CFA showed an acceptable fit to the data: χ^2^ (372) = 1065.633, *p* < 0.001; CFI = 0.938; TLI = 0.932; RMSEA = 0.075 (90% CI 0.070–0.080); and SRMR = 0.055. As highlighted in Table 1, the three latent factors were well-defined, with standardized loadings ranging from 0.491 to 0.856. The inter-factor correlations were below 0.80, supporting the distinctiveness of the latent factors [44]: Social Engagement with Compassion (r = 0.631, *p* < 0.001); Social Engagement with Bodily Sensations (r = 0.573, *p* < 0.001); and Compassion with Bodily Sensations (r = 0.482, *p* < 0.001). To further support the discriminant validity of the emerging factors, an alternative one-factor model was estimated, showing a very poor fit to the observed data: χ^2^ (375) = 3293.037, *p* < 0.001; CFI = 0.739; TLI = 0.717; RMSEA = 0.153 (90% CI 0.148–0.158); and SRMR = 0.112. Moreover, competitive models were tested, including a two-factor model combining the Social Engagement and Compassion factors, a two-factor model combining the Social Engagement and Bodily Sensations factors, and a two-factor model combining the Compassion and Bodily Sensations factors. None of these models showed a satisfactory fit to the data, with fit indices exceeding the recommended cut-offs for acceptable model fit [26,49].

To examine the stability and replicability of the proposed solution, the factorial structure was cross-validated on the second subsample, specifying the same pattern of correlated errors. The CFA model demonstrated a satisfactory fit to the observed data: χ^2^ (372) = 1027.793, *p* < 0.001; CFI = 0.950; TLI = 0.946; RMSEA = 0.073 (90% CI 0.068–0.078); and SRMR = 0.060. As shown in Table 2, all items exhibited salient standardized loadings on the intended dimension (>0.32; [60]), ranging from 0.353 to 0.900. The inter-factor correlations were lower than 0.80, supporting the distinctiveness of the latent factors [44]: Social Engagement with Compassion (r = 0.526, *p* < 0.001); Social Engagement with Bodily Sensations (r = 0.488, *p* < 0.001); and Compassion with Bodily Sensations (r = 0.302, *p* < 0.001). Consistently, the competitive one-factor model showed a very poor fit to the observed data, providing compelling evidence regarding the discriminant validity of the posited factors: χ^2^ (375) = 4167.891, *p* < 0.001; CFI = 0.814; TLI = 0.690; RMSEA = 0.175 (90% CI 0.170–0.180); and SRMR = 0.143.

### 3.3. Reliability and Validity

To maximize the information available from the data [61], reliability and validity evidence was gathered on the overall sample. Since coefficient alpha may produce inaccurate estimates of internal consistency when the solution does not conform to tau-equivalence [52], omega coefficients using Green and Yang’s [51] formula were computed. The omega estimates corresponding to the subscale scores were excellent: Social Engagement (ω_cat_ = 0.918), Compassion (ω_cat_ = 0.879), and Bodily Sensations (ω_cat_ = 0.918).

Zero-order correlations revealed significant associations between NPSS subscales and CEAS, SOCS-O, and BPQ, providing construct validity evidence (see Table 3). More specifically, the results showed that Social Engagement was positively correlated with the CEAS Engagement subscale (r = 0.428; *p* < 0.001); Compassion was positively associated with the SOCS total score (r = 0.450; *p* < 0.001); and Bodily Sensations was negatively associated with the BPQ Awareness subscale (r = −0.252, *p* < 0.001).

### 3.4. Gender Factorial Invariance

Factorial invariance tests were conducted on the whole sample to test the generalisability of the measurement model across genders (see Table 4). The configural invariance model exhibited a satisfactory fit to the data: χ^2^ (744) = 2098.920, *p* < 0.001; CFI = 0.941; TLI = 0.936; RMSEA = 0.074 (90% CI 0.070–0.078); and SRMR = 0.057. Afterward, invariance constraints on factor loadings (ΔRMSEA = −0.002; ΔCFI = 0.002) and items’ thresholds (ΔRMSEA = −0.004; ΔCFI = 0) were imposed, resulting in negligible changes in goodness-of-fit indices [55,56]. After establishing scalar invariance, latent mean comparisons were conducted to examine gender differences in the NPSS facets. Factor means were fixed to zero for females and freely estimated for males. Specifically, males showed higher levels of Social Engagement (Cohen’s d = 0.159, *p* = 0.048) and Bodily Sensations (Cohen’s d = 0.259, *p* = 0.002), as well as lower levels of Compassion (Cohen’s d = −0.203, *p* = 0.023), compared to females.

## 4. Discussion

The primary objectives of the present study were: (a) to evaluate the robustness and replicability of the factorial structure of the NPSS; (b) to assess the invariance of the measurement model across genders; and (c) to provide further evidence of construct validity of the scores within a large Italian community sample.

Through a cross-validation approach [32], our findings confirm the factorial validity of the NPSS. Initially, the three-factor model proposed by Morton and colleagues [24] did not fit the data satisfactorily in a randomly split subsample. However, through a careful examination of the modification indices and a consideration of item content, we identified correlated residuals between certain items supported by conceptual and methodological sources of overlap above and beyond the common factor. After freely estimating plausible error covariances between two pairs of items, the revised three-factor model demonstrated satisfactory fit indices in both the calibration and validation samples. The three latent factors, labeled *Social Engagement*, *Compassion*, *and Bodily Sensations*, maintained empirical distinctiveness, as evidenced by inter-factor correlations below 0.80 and by the alternative one-factor model, which showed a very poor fit to the data (i.e., Harman’s single-factor test). Importantly, the cross-validation approach supports the stability of the revised factorial structure across the two random subsamples. Moreover, reliability analyses through omega coefficients indicated excellent internal consistency for each subscale, aligning with findings from Morton et al. [24] and by Poli and Miccoli [31].

The NPSS also exhibited factorial invariance across genders. This means that the three latent dimensions of the NPSS hold consistent meaning for both males and females, enabling an accurate interpretation of gender differences. Notably, latent mean comparisons revealed small but significant gender differences in the facets of psychological safety. Specifically, males reported higher levels of Social Engagement and Bodily Sensations, while females reported higher levels of Compassion. These gender differences reflect variations in the neuroceptive processes underlying psychological safety and are in line with previous literature showing that females showed higher levels of compassion toward others [27] and poorer interoceptive accuracy compared to males [28].

While prior studies have shown that males tend to have higher levels of self-compassion [62], this study is the first to provide evidence that males reported higher levels of social engagement. These findings, in line with the tendency of personalized medicine and with research consistently reporting gender differences in mental health [29,30], suggest that interventions should take into account gender differences. Tailoring programs to evaluate how males and females differently experience social engagement, compassion, and bodily sensations could enhance the effectiveness of these programs, particularly in educational and therapeutic settings.

To establish the construct validity of the scale, the NPSS subscales were correlated with external variables within the same nomological network. The Social Engagement factor of the NPSS strongly and positively correlated with the Engagement subscale of the CEAS. This finding aligns with our expectations, as both scales assess the perception of compassion from others. More specifically, Social Engagement as conceptualized by Porges [3] involves evaluating the social environment as safe, not hazardous, experiencing acceptance, and having the freedom to express oneself without judgment [24]. This dimension reflects the perception to be trusted, understood, welcomed, and cared for. Similarly, the CEAS Engagement measures the perceived compassion from others when expressing one’s feelings of distress [6]. Our findings also showed a strong positive correlation between the Compassion of the NPSS and the total score of the SOCS_O, reflecting the ability to perceive and respond to others’ distress, consistent with the polyvagal theory’s emphasis on connection, empathy, and compassion [3]. Polyvagal theory suggests that detecting safety through neuroception activates physiological, emotional, and cognitive processes that foster compassion toward and from others, supported by the ventral vagal parasympathetic pathway [10,63]. This pathway is crucial for emotional and cognitive processes that underpin effective social interactions and bonding. Consistently, compassion and psychological safety are intricately linked, with compassion serving as a means of communicating safety [3,6]. Consistently, previous research has demonstrated that feeling validated or understood has significant psychological and physiological benefits [64]. The capacity to empathize, the ability to give or receive compassion, and positive social interactions are associated with higher levels of well-being and healthy psychological functioning [65,66,67,68]. Social safety, which engages the parasympathetic nervous system (PNS), in turn enhances social engagement behaviors (e.g., reciprocal eye contact and facial affect expressivity), creating a virtuous circle. Measuring these dimensions of psychological safety can, therefore, serve as an indicator of the extent to which individuals experience a supportive and non-threatening social environment. In this vein, we can gain insights into how well individuals feel connected and safe within their social contexts, which in turn can inform interventions aimed at enhancing relational dynamics and psychological safety in various settings.

Additionally, our findings also highlight a moderate negative correlation between the Bodily Sensations dimension of the NPSS and the Awareness subscale of the BPQ. While both scales assess body perceptions, the NPSS Body Sensations factor focuses on sensations of being in a state of calm through body feelings of relaxation, steady heartbeat, and breath, which are typical of the Social Engagement System activation. In contrast, the BPQ Awareness dimension evaluates the perception of threatening or negative bodily stimuli associated with sympathetic/dorsal vagal responses. The Bodily Sensations factor reflects awareness of internal physiological states, which polyvagal theory considers crucial for self-regulation and safety. This awareness is linked to interoception—the internal perception of physiological signals—which is essential for adaptive stress responses and maintaining homeostasis. Detecting and understanding these sensations can enhance one’s emotional regulation and adaptive responses to social environments. Measuring the Bodily Sensations dimension of psychological safety is important for understanding how individuals perceive and regulate their internal physiological states in relation to their sense of safety and well-being. By assessing Bodily Sensations, we can gain valuable insights into how physiological experiences of calm and comfort contribute to overall psychological safety. This understanding can help in designing interventions aimed at improving self-regulation, reducing stress, and enhancing emotional resilience, thereby supporting better mental health and well-being.

In summary, the NPSS subscales align well with existing theoretical constructs, demonstrating robust construct validity. By integrating psychological, relational, and physiological dimensions of safety, the scale offers valuable insights applicable across various health and social care contexts. For instance, a lack of perceived safety has been linked to an increased risk of PTSD and adverse health outcomes [69,70], and when this fundamental need is unmet, individuals often experience maladaptive consequences such as physical symptoms, depression, burnout, and negative affect [71]. Employing the NPSS to monitor shifts in the window of tolerance for autonomic arousal could significantly enhance trauma interventions [3,24]. Furthermore, understanding clients’ needs for psychological safety can aid in tailoring therapeutic approaches, thereby improving treatment outcomes. Clinically, the NPSS provides a measure to address fundamental needs that impact well-being. Focusing on positive indicators of safety rather than solely on pathological symptoms may shift trauma care approaches toward fostering environments where both patients and healthcare providers feel safe and supported [72]. Additionally, insights into neurobiological responses to stressors could help healthcare providers develop more effective interventions. Moreover, measuring psychological safety during psychotherapeutic treatments could be beneficial, as it may influence patient recovery by affecting emotional and physiological responses.

### Limitations

Importantly, several limitations of the present study should be acknowledged. First, this study did not assess the test–retest reliability of the instrument, which was recently demonstrated by Poli and Miccoli [31]. A test–retest procedure is essential for determining an estimate of the stability of the construct being evaluated, ensuring that any changes in the construct are due to genuine changes in the individual rather than a random variation or a systematic deviation due to the measurement tool. Secondly, we assessed the discriminant validity of the three NPSS factors by examining whether the three latent dimensions were empirically distinguishable. Our findings highlighted that the factors were not highly correlated, supporting the notion that they represent distinct facets [44]. However, future research should investigate whether the NPSS is uncorrelated with alternative measures with which it is supposed to differ. Similarly, it is worth noting that only self-report questionnaires were administered to examine the construct validity of the NPSS, which may suffer from social desirability and recall biases. For instance, social desirability, defined as the tendency to present oneself in a favorable manner, may confound research results by generating spurious relationships or obscuring true associations between variables [73]. Hence, future studies are needed to strengthen the validity evidence of the scale by integrating both objective and subjective measures, as well as by examining other facets of validity (e.g., nomological validity). Another significant avenue for future research is to explore the relationship between the NPSS scores and other measures of psychological well-being. Investigating how NPSS dimensions correlate with various indicators of psychological health, including life satisfaction, resilience, and overall emotional functioning, could deepen our understanding of its clinical and empirical applications. Moreover, future studies could examine how NPSS scores relate to measures of depression, anxiety, and stress and whether these relationships may differ across various populations and settings, including clinical settings, community-based programs, and educational institutions. Additionally, although factorial invariance has been established across gender, future studies may benefit from the inclusion of other demographic variables, such as marital status and education, to ascertain whether the measurement model remains consistent across these demographic groups. Finally, the present study enrolled a convenience sample, which may result in a non-representative sample of the Italian population. This limitation could affect the generalizability of the findings, as the sample might not accurately reflect the broader population’s characteristics in terms of age, gender, socioeconomic status, or other relevant factors. Future replication studies are warranted to foster the external validity of the investigation and to cross-validate the factorial structure of the scale.

## 5. Conclusions

Despite the aforementioned limitations, our study presents several strengths. Firstly, we adopted a cross-validation approach for examining the factorial validity of the scale, thereby enhancing the robustness and generalizability of our findings. Secondly, we provided evidence of construct validity and demonstrated the empirical distinctiveness of the three NPSS latent dimensions. Thirdly, our study stands as the first to assess the gender factorial invariance of the scale. Overall, our research provides compelling evidence regarding the psychometric properties of the NPSS—the first scale designed to capture various facets of psychological safety—suggesting that NPSS scores could be considered valid and reliable within the Italian context. By adapting a psychometrically sound tool assessing psychological safety, this study makes a meaningful contribution to the field, enabling researchers and practitioners to reliably assess a construct increasingly recognized as essential for mental health, well-being, and social functioning. The NPSS has the potential to inform interventions aimed at fostering environments of psychological safety, with broad applications across clinical, educational, and organizational settings. Future studies can expand on our findings by further examining the psychometric properties of the NPSS across different cultural contexts, enhancing its utility and relevance in understanding and promoting psychological safety.

## Figures and Tables

**Table 1:** Socio-demographic characteristics of the sample.

Variables	M ± SD or N (%)
**Age**	35.54 ± 14.63
**Gender**	
Female	385 (58.3%)
Male	275 (41.7%)
**Marital Status**	
Single	371 (56.2%)
Married	189 (28.6%)
Engaged in a relationship	56 (8.5%)
Divorced	35 (5.2%)
Widowed	5 (0.8%)
**Education**	
No education	3 (0.5%)
Middle school diploma	47 (7.1%)
High school diploma	286 (43.3%)
Bachelor Degree	159 (24.1%)
Master’s Degree	141 (21.4%)
Postgraduate degree	24 (3.6%)

**Table 2:** Factor loadings in a completely standardized metric for three-factor CFA solution—random samples 1 and 2.

	Random Sample 1			Random Sample 2		
	Social Engagement	Compassion	Bodily Sensations	Social Engagement	Compassion	Bodily Sensations
NPSS_1	0.752			0.761		
NPSS_2	0.751			0.707		
NPSS_3	0.775			0.770		
NPSS_4	0.750			0.780		
NPSS_5	0.789			0.728		
NPSS_6	0.817			0.757		
NPSS_7	0.608			0.725		
NPSS_8	0.642			0.693		
NPSS_9	0.793			0.784		
NPSS_10	0.835			0.843		
NPSS_11	0.773			0.791		
NPSS_12	0.761			0.784		
NPSS_13	0.741			0.769		
NPSS_14	0.491			0.353		
NPSS_15		0.785			0.734	
NPSS_16		0.799			0.800	
NPSS_17		0.584			0.570	
NPSS_18		0.696			0.725	
NPSS_19		0.844			0.870	
NPSS_20		0.804			0.824	
NPSS_21		0.744			0.847	
NPSS_22			0.775			0.830
NPSS_23			0.842			0.841
NPSS_24			0.804			0.802
NPSS_25			0.811			0.817
NPSS_26			0.774			0.716
NPSS_27			0.856			0.900
NPSS_28			0.720			0.663
NPSS_29			0.788			0.860

Note: All loadings are statistically significant (*p* < 0.001).

**Table 3:** Descriptive statistics of the NPSS and zero-order correlations with theoretically related constructs.

	Mean (SD)	SOCS_Total	CEAS_Engagement	BPQ_Awareness
NPSS_Social Engagement	50.95 (10.01)	0.197 **	0.428 **	−0.068
NPSS_Compassion	26.82 (5.26)	0.450 **	0.192 **	0.003
NPSS_Bodily Sensations	28.19 (6.90)	0.102 *	0.223 **	−0.252 **

Note: Analyses were conducted on the whole sample. * *p* < 0.01; ** *p* < 0.001.

**Table 4:** Factorial invariance tests across gender.

Model	WLSMV χ^2^ (df)	RMSEA	CFI	TLI	SRMR	Model Comparison	ΔRMSEA	ΔCFI
1. Configural invariance	2098.920 (744)	0.074	0.941	0.936	0.057			
2. Metric invariance	2080.198 (770)	0.072	0.943	0.940	0.057	2 vs. 1	−0.002	0.002
3. Scalar invariance	2178.972 (854)	0.068	0.943	0.946	0.058	3 vs. 2	−0.004	0

Note: Analyses were conducted on the whole sample.

## Data Availability

The data that support the findings of this study are available on request from the corresponding author.

## Supplementary Materials

The following supporting information can be downloaded at: https://www.mdpi.com/article/10.3390/ejihpe14100178/s1, Table S1: Item-level descriptive statistics of the NPSS.
