# Supplementary material for: Factor Structure, Construct Validity, and Measurement Invariance of the Neuroception of Psychological Safety Scale (NPSS)
Source: Eur J Investig Health Psychol Educ. 2024 Oct 4;14(10):2702–15. doi: 10.3390/ejihpe14100178 (PMC11507099; doi:10.3390/ejihpe14100178)
Supplement: Supplementary file 1 [file ejihpe-14-00178-s001.zip › ejihpe-3163521-supplementary.pdf]

Item	Mean	SD	Range
NPSS_1	3.67	0.98	1-5
NPSS_2	3.64	1.02	1-5
NPSS_3	3.75	0.93	1-5
NPSS_4	3.47	1.01	1-5
NPSS_5	3.43	0.97	1-5
NPSS_6	3.72	0.97	1-5
NPSS_7	3.93	1.04	1-5
NPSS_8	4.15	0.93	1-5
NPSS_9	3.61	1.02	1-5
NPSS_10	3.64	0.97	1-5
NPSS_11	3.50	1.05	1-5
NPSS_12	3.55	1.09	1-5
NPSS_13	3.72	1.08	1-5
NPSS_14	3.23	1.14	1-5
NPSS_15	3.95	0.95	1-5
NPSS_16	4.02	0.95	1-5
NPSS_17	3.73	1.02	1-5
NPSS_18	3.69	1.06	1-5
NPSS_19	4.06	0.96	1-5
NPSS_20	3.59	1.06	1-5
NPSS_21	3.78	0.99	1-5
NPSS_22	3.60	1.06	1-5
NPSS_23	3.62	1.15	1-5
NPSS_24	3.80	1.01	1-5
NPSS_25	3.31	1.14	1-5
NPSS_26	3.22	1.19	1-5
NPSS_27	3.72	1.05	1-5
NPSS_28	3.56	1.15	1-5
NPSS_29	3.37	1.09	1-5

Supplementary Table S1. Item-level descriptive statistics of the NPSS. Abbreviations: SD, standard deviation.
